# Comparisons of *WUE* in twelve genotypes of winter wheat and the relationship between *δ*^13^C and *WUE*

**DOI:** 10.7717/peerj.6767

**Published:** 2019-04-17

**Authors:** Guirong Huang, Xinying Zhang, Yajing Wang, Fu Feng, Xurong Mei, Xiuli Zhong

**Affiliations:** Chinese Academy of Agricultural Sciences, Institute of Environment and Sustainable Development in Agriculture, Beijing, PR China

**Keywords:** Water use efficiency, Grain yield, Stable carbon isotope composition, Winter wheat genotypes, Grain filling stage

## Abstract

Twelve winter wheat (Triticum aestivum) genotypes were examined for differences in grain yield, water use efficiency (WUE), and stable carbon isotope composition (δ13C) in flag leaves. The plants were subjected to rain-fed treatment and supplemental irrigation at the jointing and anthesis stages, during the 2015–2016 and 2016–2017 winter wheat growing seasons. The relationships between δ13C with grain yield and WUE were analyzed under two different water environments. The results indicated that there were significant differences in *δ*13C, grain yield, and WUE among wheat genotypes both under rain-fed and supplemental irrigation conditions. The δ13C values increased with grain-filling proceeding, the δ13C being lower under supplemental irrigation treatment than that under rain-fed treatment. The relationships between the average of δ13C with grain yield and WUE were significantly positive during three measurement periods (*R*2 = 0.5785 − 0.8258), whether under rain-fed or irrigation environments. This suggests that δ13C might be associated with the grain yield and WUE in winter wheat under rain-fed and supplemental irrigation conditions in the climate region of the northwest Huang-Huai-Hai Plain of China.

## Introduction

More than half of the winter wheat in China is supplied by the Huang-Huai-Hai Plain of China, which covers a 32,000 km^2^ area (Zhang et al., 2010). As the main winter wheat production area in China, the Huang-Huai-Hai Plain does not have consistent annual rainfall, with less than 200 mm of rainfall, but more than 400 mm of evapotranspiration during the winter wheat growing season (Li et al., 2012; Fulvia et al., 2012). Moreover, climate change is forecasted to further compromise the water quality and availability for supply (Sowers, Vengosh & Weinthal, 2011; Garcia & Pargament, 2015). The Gravity Recovery and Climate Experiment (GRACE) detected a significant groundwater storage (GWS) depletion rate of 7.2 ± km^3^/yr in the Huang-Huai-Hai Plain of China during 2002–2014 (Feng et al., 2018). New drought-resistant and water-saving cultivars are needed to ensure production capacity under climate change scenarios. Water use efficiency (*WUE*) represents the relationship between carbon assimilation and water used. Thus it is vital to increase *WUE* for sustaining wheat production with the increasing water scarcity and drought trends (Bacon, 2004).

There are several scales of *WUE*. The intrinsic *WUE* is the ratio of the photosynthesis rate divided by the stomatal conductance. The leaf *WUE* or leaf transpiration efficiency (*TE*) is the ratio of the photosynthesis rate divided by transpiration rate. The *WUE* at the individual plant level is the ratio of biomass or economic weight divided by water transpired. The field *WUE* is the economic product or biomass divided by water used, including transpiration and evaporation (Mei et al., 2013).

Carbon is an important life element, and there are two stable forms (^13^C and ^12^C) in nature. Due to the difference in physical and chemical properties of ^13^C and ^12^C, isotope fractionation occurs in plants during the process of assimilating CO_2_ (Farquhar, Ehleringer & Hubick, 1989), and the stable isotope composition before and after the reaction are different. Carbon isotope composition (δ) and carbon isotope discrimination (△) are parameters that are utilized to characterize carbon isotope discrimination in plants (Xu et al., 2007). Because of the large differences in δ^13^C among natural plants (Paneth & O’Leary, 1985), δ^13^C can integrate the long-term photosynthetic, various physiological, and morphological characteristics of plants. δ^13^C is widely used in ecology, agronomy, and global change research with the advantages of simplicity, rapidity, and accuracy. For it analyzes the carbon accumulated in the leaves over a long period of time, *δ*^13^C is then used to evaluate the characteristics of *WUE* in leaves and plants during growth (Damesin & Learge, 2003; Scartazza, Mata & Matteucci, 2004).

Over the past forty years, many researchers have found that plant-stable carbon isotope composition (δ^13^C) is rather useful. In recent years, there have been many studies that used δ^13^C in plant leaves to indicate *WUE*, and some important conclusions have been made. A large number of studies have shown that there is a significant correlation between δ^13^C value with crop yield and *WUE*, and the δ^13^C in plant leaves can be the indicator of plant long-term *WUE* (Farquhar & Richards, 1984; Hubick & Farquhar, 1987; Sarange et al., 1999; McDowell, Brooks & Fitzgerald, 2003). Studies have found that δ^13^C is heritable (Condon & Richards, 1992). Simultaneously, grain yield is positively associated with △ in grain at maturity and in leaves at anthesis (Xu et al., 2007). The stage of sampling, the analyzed organ or tissue, and the water regime can affect the correlation between △ and grain yield (Xu et al., 2007).

The highest negative correlations between δ^13^C in mature grains and grain yield across durum wheat genotypes were shown under rain-fed and irrigation conditions. While the flag leaf, δ^13^C was negatively correlated with grain yield only under rain-fed conditions (Bort et al., 2014). In addition, leaf *WUE* was affected by the ratio of CO_2_ concentration in the leaves and CO_2_ concentration in the atmosphere (Ci/Ca), while the *δ*^13^C values were closely related to Ci/Ca (Ehleringer, 1993). This suggested that there may be some relationships between δ^13^C value with the grain yield and *WUE*. Studies on grain *WUE* could be conducted to supply guidance for agricultural practices on the North China Plain (Mei et al., 2013). However, there are some arguments on the stability of relationships of △^13^C in various tissues with yield and *WUE*, especially concerning crop *WUE* on the field scale (Anyia et al., 2007; Seibt et al., 2008; Yousfi, Serret & Araus, 2013). TE on individual plant scale and effective amounts of water used for production have no correlation with △^13^C using isogenic materials with large variation in TE (Devi et al., 2011).

Furthermore, the selection criteria for grain yield and *WUE* are important aspects of water-saving agricultural research. A large number of studies have been carried out from the aspects of morphology, physiology, and molecular biology, aiming at finding out reliable identification indicators for selecting drought-resistant and water-saving crop genotypes. To date, however, there are not many rapid, simple screening methods that will assist mass selection in breeding programs.

Although field *WUE* provides the most meaningful guidance for agricultural practices, it is laborious and time consuming, thus not suitable for evaluation of a large number of varieties. Essentially, the data on the correlation between leaf *δ*^13^C concentrated on different grain-filling stages, grain yield, and *WUE* on a field scale are lacking. In our experiment, 12 different genotypes of winter wheat with different morphological and physiological characteristics were selected for study. The δ^13^C values in the flag leaves of different genotypes and their relationships with yield and *WUE* were studied under different water conditions with rain-fed and supplemental irrigation at the jointing and anthesis stages to provide a basis for screening drought-resistant and water-saving varieties.

## Materials and Methods

### Experimental site

The experiment was carried out at the Shunyi Experimental Station (20 m above sea level, 40°N and 116°E), located in the northern part of the Huang (Yellow River)-Huai (Huai River)-Hai (Hai River) Plain of China. The area is in a warm temperate semi-humid continental monsoon climate zone with most of annual rainfall falling during the summer season. In this region, the mean rainfall is 625 mm, of which approximately 75% falls between July and August, and the annual average temperature is 11.5 °C, with average annual sunshine of 2,750 h. The experiment was carried out in the 2015–2016 and 2016–2017 winter wheat growing seasons. Each experimental plot was 10.0 m ×2.4 m, and the plot consisted of 12 lines separated by 20 cm. The levels of organic matter, total nitrogen, rapidly available phosphorus, rapidly available potassium, and pH in a 0–20 cm soil layer were 14.4 g kg^−1^, 1.1 g kg^−1^, 24.5 mg kg^−1^, 10 mg kg^−1^, and 7.7, respectively; 112.5 kg ha^−1^ of nitrogen, 75 kg ha^−1^ of phosphorus pentoxide, and 75 kg ha^−1^ of potassium oxide were present, and the previously planted crop was field peas (Pisumsativum spp. arvense). The winter wheat was planted at a density of 6.75 × 10^6^ plants ha^−1^ on October 5, 2015 and October 3, 2016. Winter wheat was harvested on June 12, 2016 and June 11, 2017. Eight near isogenic lines (NILs) along with their donor parent Jing 411 and recurrent parent Jinmai 47 and Shijiazhuang 8 and Yumai 18 were adopted as materials in this experiment. Twelve winter wheat genotypes have different drought-resistant and water-saving characteristics.

**Figure 1:**
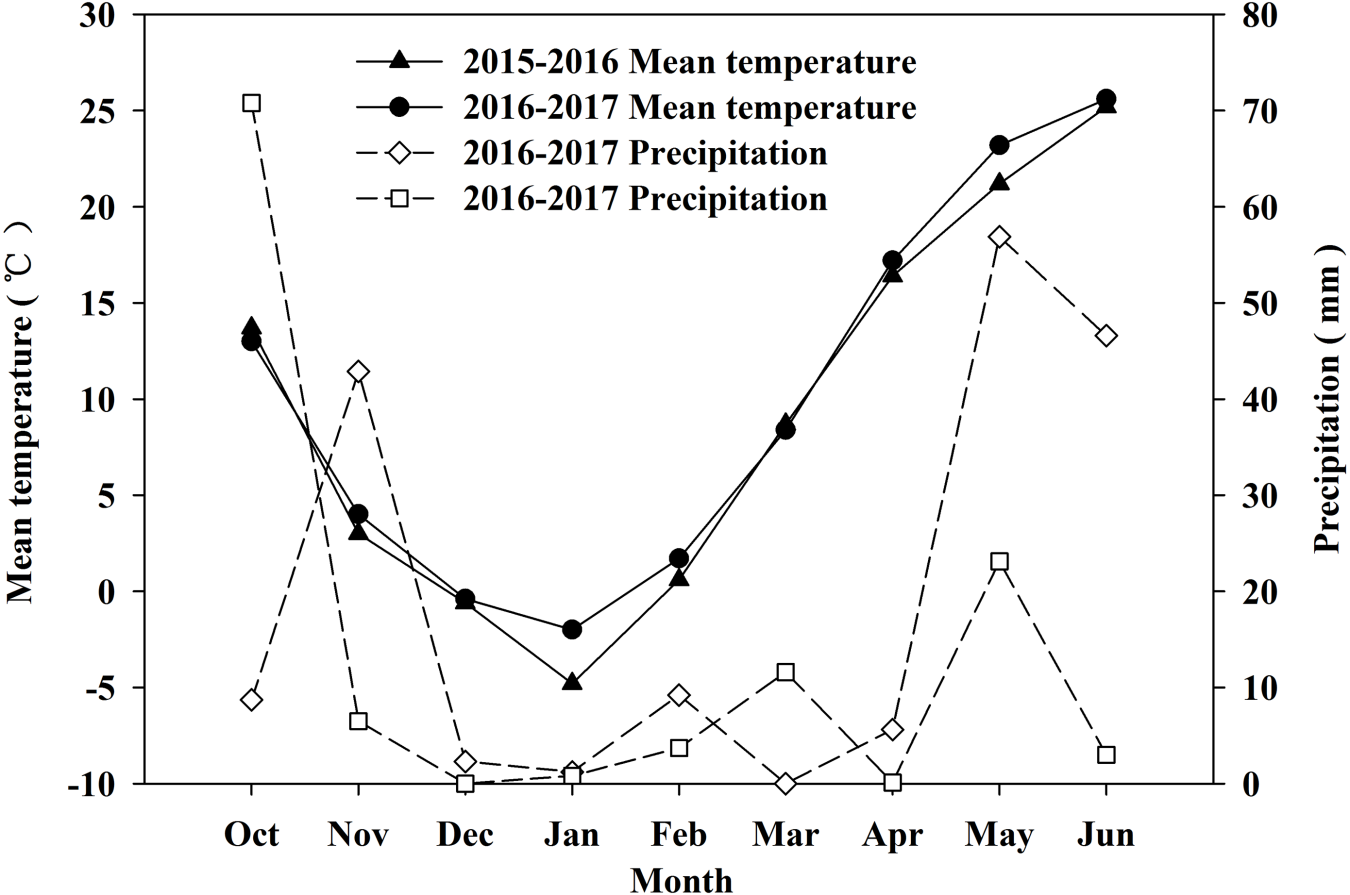
Monthly precipitation and temperature during 2015–2016 and 2016–2017 winter wheat growing seasons. Precipitation in October was sum from sown day to the last day of October. Precipitation in June was sum from June 1 to harvested day.

In the 2015–2016 and 2016–2017 winter wheat growing seasons, the precipitation totals were 173.4 and 119.6 mm, respectively (Fig. 1), which were less than the average seasonal precipitation (242.6 mm ) by 69.2 and 123 mm, respectively. In the 2015–2016 winter wheat growing season, precipitation occurred mainly from April to June, accounting for 62.9% of the total annual precipitation. By contrast, during the 2016–2017 winter wheat growing season, precipitation occurred mainly from October to November, and the precipitation during this period accounted for 64.4% of the total annual precipitation.

### Experimental design

Twelve winter wheat genotypes, which differ in population water use efficiency, were selected as materials, based on the previous studies regarding evaluation of water consumption traits. Rain-fed and supplemental irrigation conditions were set as two water treatments. For rain-fed treatments, no irrigation was conducted after sowing in the winter wheat growing season. For supplemental irrigation treatments, 60 mm water was applied to each plot at the jointing and flowering stages by sprinkling water with a flow meter to record the irrigated water amount. Before sowing, each plot of the two treatments was watered uniformly to field capacity to ensure that the seeds would sprout and grow into intact seedlings. Each experimental growing plot was 10m in length and 2m in width. The plots were arranged in randomized blocks with three replications. Soil moisture content was measured at the sowing stage and final harvest stage, grain yield was determined after harvest, and leaf samples were taken in early, middle, and late grain filling stages for measurement of δ^13^C.

### Measurements

#### Soil moisture content

The soil moisture content obtained at every 20 cm down to 160 cm of soil was measured by the oven-drying method (Li et al., 2015). Measurements were performed at the sowing stage and final harvest stage. The soil sample is placed immediately in an aluminum box after it is taken. The fresh weight is placed in a drying oven and dried at 105 °C for 24 h to constant weight. The dried soil is weighed and the soil moisture content is calculated. Soil moisture content=(fresh soil weight-dry soil weight)/dry soil weight ×100%.

#### Grain yield

When the winter wheat had attained maturity, 1-m lengths of 5 rows were manually harvested at random in every experimental plot and air dried.

#### Evapotranspiration

Evapotranspiration (ET) was calculated using the soil water balance equation (Li et al., 2012):

(1) \begin{eqnarray*}\text{ET}=\text{I}+\text{P}-\text{R}-\text{D}-\text{SW},\end{eqnarray*}

where ET denotes evapotranspiration (mm), I denotes the irrigation amount (mm), P denotes precipitation (mm) that was measured by the weather station at the site, R denotes the surface runoff (mm) and runoff was not observed due to the low precipitation, D denotes the downward drainage below the crop root zone (mm), and it was negligible because the soil moisture measurements indicated that drainage at the experimental site was negligible, and therefore, deep percolation was ignored, and SW denotes the change in water storage in the soil profile exploited (0–160 cm in depth) from planting to harvest by crop roots (mm).

#### Water use efficiency

Water use efficiency (WUE) in relation to the grain yield is defined as follows (Sadras & Lawson, 2013):

(2) \begin{eqnarray*}\mathrm{WUE}= \frac{\text{Y}}{\text{ET}} ,\end{eqnarray*}

where Y (kg ha^−1^) denotes the winter wheat grain yield, and ET (mm) denotes the total evapotranspiration during the entire winter wheat growing season derived from Eq. (1).

#### Sample collection

Wheat leaf samples were taken on May 13, 22, and June 1, 2016, and May 11, 20, and 29, 2017, corresponding to early, middle, and late stage of wheat grain filling. 10-15 fully expanded flag leaves from 5 spots in each plot were taken and pooled. A portion of this pooled sample was then analyzed.

#### Measurement of stable carbon isotope composition (*δ*^13^C)

The samples were placed in paper envelopes and brought back to the laboratory, and then, they were dried to a constant weight at 70 °C, crushed, and sieved through 200 mesh. δ^13^C was determined using a combination of a Vario PYRO cube element analyzer and ISOPRIME-100 stable isotope mass spectrometer (Elemental Microanalysis, Okehampton, UK). The standard substance used for sample determination was a Low Organic Soil Standard, standard value :*δ*^13^Cv-PDB: −26.66, C%: 1.61. In the analysis process, every 12 samples were interspersed with a laboratory standard for calibration. The long-term standard deviation of the instrument was 0.2‰, and C is a calibration result based on the international standard V-PDB.

#### Statistical analysis

The collected data for all of the measurements were statistically analyzed with three-factor ANOVA to test the difference among wheat genotypes and between two water treatments, using SAS software (version 9.2; SAS Institute, Cary, NC, USA). The multiple comparisons were performed at *α* = 0.05 level of significance with LSD test. The Cluster Procedure (Single Linkage Cluster Analysis) has been used for factors. Correlation analysis was conducted to relate the δ^13^C with grain yield and *WUE*.

## Results

### Comparison of *WUE* in 12 genotypes of winter wheat

**Figure 2:**
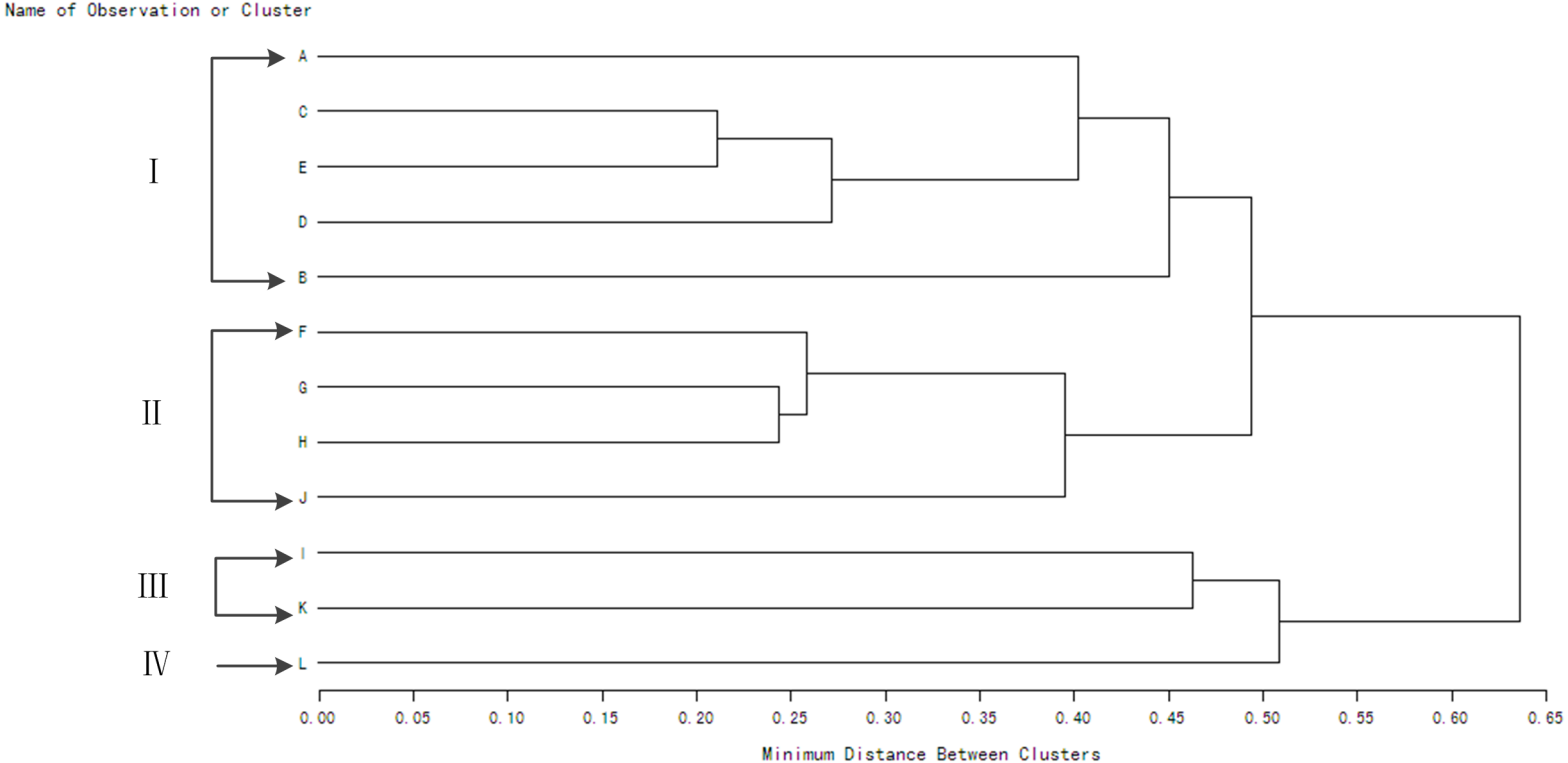
Clustering dendrogram of winter wheat genotypes based on WUE values for consecutive seasons under rain-fed condition. A-Jinmai 47; B-Shijiazhuang 8; C-908216; D-908032; E-908188; F-908274; G-908054; H-908206; I-908120; G-Yumai 18; K-908092; L-Jing 411.

**Figure 3:**
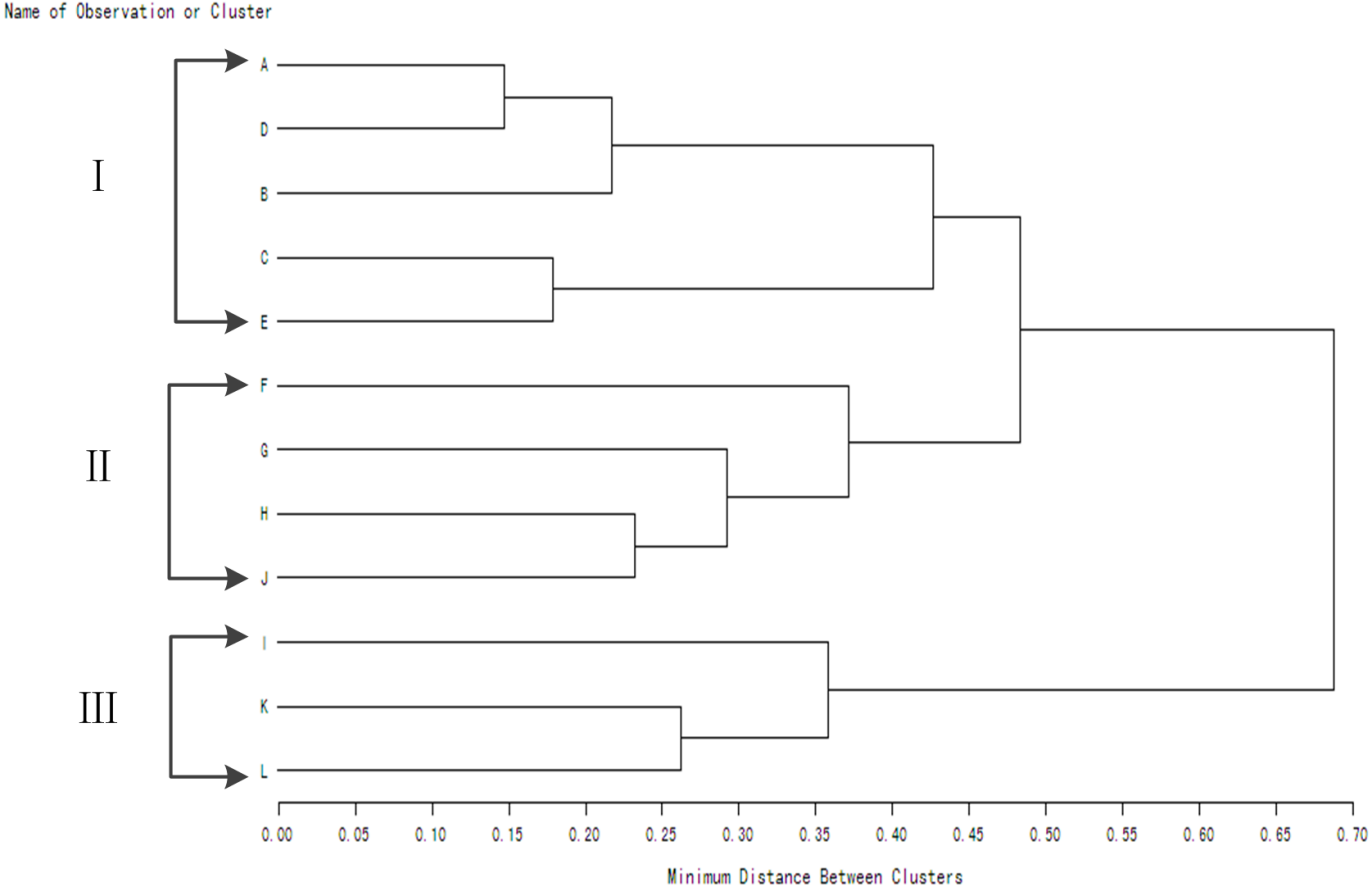
Clustering dendrogram of winter wheat genotypes based on WUE values for consecutive seasons under supplemental irrigation condition. A-Jinmai 47;B-Shijiazhuang 8;C-908216;D-908032;E-908188;F-908274;G-908054; H-908206;I-908120; G-Yumai 18;K-908092;L-Jing 411

There were significant differences in *WUE* in different wheat genotypes under the same water conditions and water condition accounting for 14.51% of the total variance together with two-factor and three-factor interaction between genotype and water condition had significant effect on *WUE* (Table 1). The method of “Minimum Distance Between Clusters” was used to generate Figs. 2 and 3. Under rain-fed condition, the materials were divided into four groups with different *WUE* (Fig. 2). There were five genotypes in group I, four genotypes in group II, two genotypes in group III, and just one genotype in group IV. The average value of *WUE* in group I was significantly higher than that in groups II, III, and IV. The genotypes in group I exhibited a higher grain yield and lower water consumption. The genotypes in group II exhibited moderate grain yield and moderate water consumption. The genotypes in groups III and IV exhibited lower grain yield and higher water consumption. Under supplemental irrigation conditions, the materials were divided into three groups with different *WUE* (Fig. 3).

**Table 1:** A three-factor ANOVA analysis for WUE.

Source of variation	Degree of freedom	Sum of squares	Proportion(%)	Mean square	*F* value	*P* value
Block (B)	2	24.488	5.6	12.244	320.49**	<0.0001
Genotype (G)	11	316.84	72.52	28.803	753.96**	<0.0001
Water condition (W)	1	63.401	14.51	63.401	1659.61**	<0.0001
Year (Y)	1	0.0023	0	0.0023	0.06	0.8085
G × W	11	7.6021	1.74	0.6911	18.09**	<0.0001
G × Y	11	12.643	2.89	1.1494	30.09**	<0.0001
W × Y	1	0.5968	0.14	0.5968	15.62**	0.0001
G ×W × Y	11	11.345	2.6	1.0313	27**	<0.0001

**Notes.**

Proportion can be achieved by dividing the sum of squares of each factor by the total sum of squares.

Both water consumption and grain yield in winter wheat genotypes were significantly different regardless of rain-fed or irrigation treatment (Tables 2–3). Water condition had the largest effect on grain yield and water consumption compared with other factors and interactions. The average grain yield difference between two water conditions was 1,592 kg ha^−1^ in 2015–2016, and 1,563 kg ha^−1^ in 2016–2017 growing seasons, respectively. However, genotypes differed largely in response to soil moisture alteration (Tables 4–5). The largest increase in grain yield occurred in genotype ‘908032’ with 1,925 kg ha^−1^, and the smallest in genotype ‘908188’ with 1,267 kg ha^−1^ in 2015–2016. The largest increase in grain yield was in genotype ‘908120’ with 2,494 kg ha^−1^, and the smallest in genotype ‘908274’ with 835 kg ha^−1^ in 2016–2017 growing seasons. This implied different responses to supplemental irrigation applied at jointing and anthesis stages, which brought about ultimate different yield responses, among wheat genotypes. The variation of the *WUE* among 12 genotypes was basically consistent with the grain yield. Under irrigation treatment, the average *WUE* for wheat genotypes was higher than that under rain-fed conditions by 1.5 kg ha^−1^ mm^−1^ in 2015–2016 growing season, and by 1.2 kg ha^−1^ mm^−1^ in 2016–2017 growing season, respectively. Similarly, genotypes differed largely in *WUE* increase under irrigation treatment, compared with rain-fed condition. The largest increase was in genotype ‘908032’ at 3.07 kg ha^−1^ mm^−1^, and the smallest increase was in genotype ‘908188’ at 0.13 kg ha^−1^ mm^−1^ in 2015–2016 growing season. The largest increase was in genotype ‘908188’ at 2.43 kg ha^−1^ mm^−1^ and the smallest increase was in genotype ‘Shijiazhuang 8’ at 0.3 kg ha^−1^ mm^−1^ in 2016–2017 growing season. Also, with the same amount of water consumption, genotypes differed significantly in grain yield. These results comprehensively reflected the differences in response to supplemental irrigation during critical water demand stages among genotypes. Moreover, under rain-fed and supplemental irrigation conditions, the grain yields were significantly and linearly positively correlated with the *WUE* (*R*^2^ = 0.8231 − 0.9375).

**Table 2:** A three-factor ANOVA analysis for grain yield.

Source of variation	Degree of freedom	Sum of squares	Proportion(%)	Mean square	*F* value	*P* value
Block (B)	2	2957077	2.29	1478538	76.04**	<0.0001
Genotype (G)	11	30625071	23.76	2784097	143.18**	<0.0001
Water condition (W)	1	89624358	69.54	89624385	4609.09**	<0.0001
Year (Y)	1	954932	0.74	954932	49.11**	<0.0001
G × W	11	1292098	1	117463	6.04**	<0.0001
G × Y	11	1644491	1.28	149499.2	7.69**	<0.0001
W × Y	1	7706.52	0.01	7706.52	0.4	0.5305
G ×W × Y	11	1772610	1.38	161146.4	8.29**	<0.0001

**Notes.**

Proportion can be achieved by dividing the sum of squares of each factor by the total sum of squares.

**Table 3:** A three-factor ANOVA analysis for water consumption.

Source of variation	Degree of freedom	Sum of squares	Proportion(%)	Mean square	*F* value	*P* value
Block (B)	2	661.4279	0.38	330.7139	10.15**	0.0001
Genotype (G)	11	15492.26	8.89	1408.388	43.22**	<0.0001
Water condition (W)	1	145107.3	83.25	145107.3	4452.93**	<0.0001
Year (Y)	1	3089.673	1.77	3089.673	94.81**	<0.0001
G × W	11	2019.812	1.16	183.6193	5.63**	<0.0001
G × Y	11	3953.958	2.27	359.4507	11.03**	<0.0001
W × Y	1	390.0391	0.22	390.0391	11.97**	0.0008
G ×W × Y	11	3594.97	2.06	326.8155	10.03**	<0.0001

**Notes.**

Proportion can be achieved by dividing the sum of squares of each factor by the total sum of squares.

**Table 4:** Grain yield, water consumption, and water use efficiency (WUE) in 2015–2016 growing season.

Genotype	Rain-fed			Irrigation			Difference	
	Grain yield (kg ha^−1^)	Water consumption (mm)	WUE (kg ha^−1^ mm^−1^)	Grain yield (kg ha^−1^)	Water consumption (mm)	WUE (kg ha^−1^ mm^−1^)	Grain yield (kg ha^−1^)	WUE (kg ha^−1^ mm^−1^)
Jinmai 47	6597a	358.7cd	18.39a	8177a	422.9cd	19.2a	1516bcdef	1.5de
Shijiazhuang 8	6459ab	357.4cde	18.07ab	8165a	424.8bcd	19.28a	1706abcd	1.23ef
908216	6163bc	358.8cd	17.18bc	7502bc	409.3fg	18.31bc	1440cdef	0.73 g
908032	5909cd	346.2fg	17.07bc	7631b	402.1 g	18.94ab	1925a	3.07a
908188	5738de	341.9 g	16.78cd	7479bc	411.6efg	17.98cd	1267f	0.13 h
908274	5634de	348.0efg	16.19cde	6901ef	422.1cde	16.34fg	1740abc	1.2efg
908054	5614def	352.0defg	15.95de	7054de	423.4bcd	16.7ef	1426def	1.07efg
908206	5452efg	355.5def	15.34ef	7235cd	405.0fg	17.67cd	1783ab	2.3bc
908120	5384efg	366.2c	14.7fg	6809ef	430.8abc	15.75gh	1721abcd	1.87cd
Yumai 18	5213fgh	367.8bc	14.17gh	7138de	413.8def	17.23de	1339ef	1.13efg
908092	5058gh	377.8ab	13.39hi	6574f	439.9a	14.9i	1580bcde	0.83fg
Jing 411	4935 h	387.6a	12.73i	6601f	434.4ab	15.13hi	1666abcd	2.4b
Mean	5680	359.8	15.83	7272	420.0	17.29	1592	1.50
CV(%)	3.99	1.35	3.47	2.37	1.25	0.45	10.12	16.18

**Notes.**

Values without the same letters in the same line are significantly different at 5% level; Difference here indicated the gap of grain yield and WUE between irrigation and rain-fed treatments.

**Table 5:** Grain yield, water consumption, and water use efficiency (WUE) in 2016–2017 growing season.

Genotype	Rain-fed			Irrigation			Difference	
	Grain yield (kg ha^−1^)	Water consumption (mm)	WUE (kg ha^−1^ mm^−1^)	Grain yield (kg ha^−1^)	Water consumption (mm)	WUE (kg ha^−1^ mm^−1^)	Grain yield (kg ha^−1^)	WUE (kg ha^−1^ mm^−1^)
Jinmai 47	6277a	363.2b	17.28bc	7929a	425.4b	18.64a	1620b	1.9b
Shijiazhuang 8	5989b	322.8f	18.55a	7433b	393.9f	18.87a	1444bc	0.3f
908216	5743bc	330.9e	17.36bc	7321bc	406.0de	18.03ab	1604b	0.87de
908032	5481cde	330.4e	16.59cd	7974a	420.9bc	18.94a	1511bc	0.8de
908188	6401a	361.1b	17.73ab	7236bc	396.5ef	18.25ab	1754b	2.43a
908274	5250ef	350.3c	14.99fg	7004bcd	401.9def	17.43bc	835d	0.5ef
908054	5319def	341.0d	15.6ef	6923cde	419.8bc	16.49cd	1515bc	1.03cd
908206	5584cd	349.2c	15.99de	6763def	397.6ef	17.01c	1179cd	1cd
908120	4948g	360.2b	13.74hi	6463ef	437.7a	14.76e	2494a	2.37ab
Yumai 18	5466cde	337.2d	16.21de	6977bcd	410.2cd	17.01c	1577b	0.67def
908092	4835g	369.7a	13.08i	6456f	431.3ab	14.97e	1652b	1.37c
Jing 411	5085fg	351.2c	14.48gh	6657def	427.3ab	15.58de	1572b	1.1cd
Mean	5531	347.3	15.97	7095	414.0	17.17	1563	1.20
CV(%)	2.91	1.14	3.02	3.58	1.35	3.14	11.92	18.23

**Notes.**

Values without the same letters in the same line are significantly different at 5% level; Difference here indicated the gap of grain yield and WUE between irrigation and rain-fed treatments.

### Stable carbon isotope composition (*δ*^13^C) in flag leaves

The 12 winter wheat genotypes exhibited significant differences in δ^13^C values of flag leaves during early, middle, and late grain filling stages, both under rain-fed and supplemental irrigation conditions (Tables 6–7). For the three measurement time points from early to late grain filling stage, δ^13^C of some genotypes did not change substantially, while some genotypes increased significantly (*P* < 0.001) under both water conditions (Tables 6–7). The order of 12 winter wheat genotypes in term of δ^13^C values remained nearly the same at three measurements under the two water treatments, indicating the stability of δ^13^C in characterizing plants. The δ^13^C values of flag leaves were significantly higher under rain-fed conditions than that under supplemental irrigation conditions (Tables 6–7).

**Table 6:** Stable carbon isotope composition (*δ*13C) in flag leaves of different winter wheat genotypes in 2015–2016 growing season.

Genotype	*δ*^13^C value(‰)					
	Rain-fed			Irrigation		
Date (month/day)	May 12	May 21	May 30	May 12	May 21	May 30
Jinmai 47	−25.5a	−25.1a	−25a	−26.4b	−26.1a	−25.6a
Shijiazhuang 8	−26.3d	−25.9c	−25.7c	−26.6c	−26.4b	−25.9c
908216	−25.9b	−25.3b	−25.1b	−26.2a	−26.1a	−25.7b
908032	−26c	−25.9c	−25.7c	−26.8d	−26.4b	−26d
908188	−27e	−26.3d	−26.3d	−27.4 g	−27.2 g	−26.8 g
908274	−27e	−26.8f	−26.7 h	−27.1e	−27e	−26.7f
908054	−27.2 g	−27g	−26.7 h	−27.9i	−27.2 g	−27.1i
908206	−27.1f	−26.8f	−26.5f	−27.1e	−26.9d	−26.6e
908120	−27.1f	−26.7e	−26.4e	−27.3f	−27.1f	−26.7f
Yumai 18	−27.4 h	−27.2 h	−26.7 h	−27.3f	−26.7c	−26.6e
908092	−27.2 g	−27g	−26.6 g	−27.9i	−27.5i	−27.2j
Jing 411	−27.4 h	−27g	−26.7 h	−27.6 h	−27.4 h	−27 h
Mean	−26.76	−26.42	−26.18	−27.13	−26.83	−26.49
*CV* (%)	0.54	0.49	0.5	0.51	0.53	0.51

**Notes.**

Values without the same letters in the same line are significantly different at 5% level.

**Table 7:** Stable carbon isotope composition (*δ*13C) in flag leaves of different winter wheat genotypes in 2016–2017 growing season.

Genotype	*δ*^13^C value(‰)					
	Rain-fed			Irrigation		
Date (month/day)	May 11	May 20	May 29	May 11	May 20	May 29
Jinmai 47	−27.17a	−27.07a	−26.56b	−28.14b	−27.81b	−27.63b
Shijiazhuang 8	−27.32ab	−27.09a	−26.66bc	−28.07b	−27.80b	−27.60b
908216	−27.46bc	−27.28ab	−26.89cd	−28.18b	−27.81b	−27.72b
908032	−27.32ab	−27.11a	−26.89cd	−27.67a	−27.34a	−27.24a
908188	−27.16a	−27.01a	−25.93a	−28.19b	−27.84bc	−27.72b
908274	−28.12d	−27.88d	−27.22ef	−28.66c	−28.20d	−27.83b
908054	−27.56bc	−27.43bc	−26.96de	−28.58c	−28.01bcd	−27.79b
908206	−28.06d	−27.59c	−27.38fg	−28.64c	−28.07cd	−27.82b
908120	−28.24d	−28.07d	−27.64 g	−28.86c	−28.71e	−28.47c
Yumai 18	−27.69c	−27.57c	−27.43fg	−28.20b	−27.91bc	−27.75b
908092	−28.20d	−28.01d	−27.54 g	−28.85c	−28.63e	−28.35c
Jing 411	−27.65c	−27.55bc	−27.37fg	−28.21b	−27.99bcd	−27.76b
Mean	−27.66	−27.47	−27.04	−28.35	−28.01	−27.81
*CV* (%)	0.55	0.53	0.57	0.54	0.46	0.43

**Notes.**

Values without the same letters in the same line are significantly different at 5% level.

ANOVA of δ^13^C showed that the factors investigation year (Y), genotype (G), stage (S), water condition (W), and block (B) all had significant effects on *δ*^13^C (Table 8). Variance in δ^13^C from Y was the largest, covering 43.94% of the total variance. G ranked the second to Y, occupying 25.64%. Proportion of variance from S, W, and B were rather smaller than that of Y and G. This indicated that Y was the most influential variable for δ^13^C, and G had important effects on δ^13^C. Moreover, multiple-factor interactions among Y, G, W, S, and B had significant effects on δ^13^C, except two-factor interactions between S × G, S × W, and S × Y, though the proportions of variance were rather smaller, compared with that from Y and G.

**Table 8:** Multi-factor analysis of variance for stable carbon isotope composition (*δ*13C) at grain filling stage.

Source of variation	Degree of freedom	Sum of squares	Proportion(%)	Mean square	*F* value	*P* value
Block (B)	2	1.3460551	0.47	0.6730275	37.74**	<0.0001
Genotype (G)	11	73.440293	25.64	6.6763903	374.39**	<0.0001
Water condition (W)	1	28.440293	9.93	28.358978	1590.27**	<0.0001
Year (Y)	1	125.88223	43.94	125.88223	7059.04**	<0.0001
Stage (S)	2	26.320003	9.19	13.160002	737.97**	<0.0001
G × W	11	4.3441806	1.52	0.3949255	22.15**	<0.0001
G × Y	11	16.630799	5.81	1.5118909	84.78**	<0.0001
W × Y	1	2.2309313	0.78	2.2309313	125.1**	<0.0001
G × S	22	0.6095616	0.21	0.0277073	1.55	0.0565
W × S	2	0.0633839	0.02	0.0316919	1.78	0.171
Y × S	2	0.300182	0.1	0.015091	0.85	0.4301
G ×W × S	22	0.946086	0.33	0.0430039	2.41**	0.0005
G ×Y × S	22	0.8553967	0.3	0.0388817	2.18**	0.0021
W ×Y × S	2	0.3989902	0.14	0.1994951	11.19**	<0.0001
G ×W × Y	11	3.3100238	1.15	0.3009113	16.87**	<0.0001
G ×W ×Y × S	22	1.3601349	0.47	0.0618243	3.47**	<0.0001

### Relationship between the *δ*^13^C value, grain yield, and *WUE*

**Figure 4:**
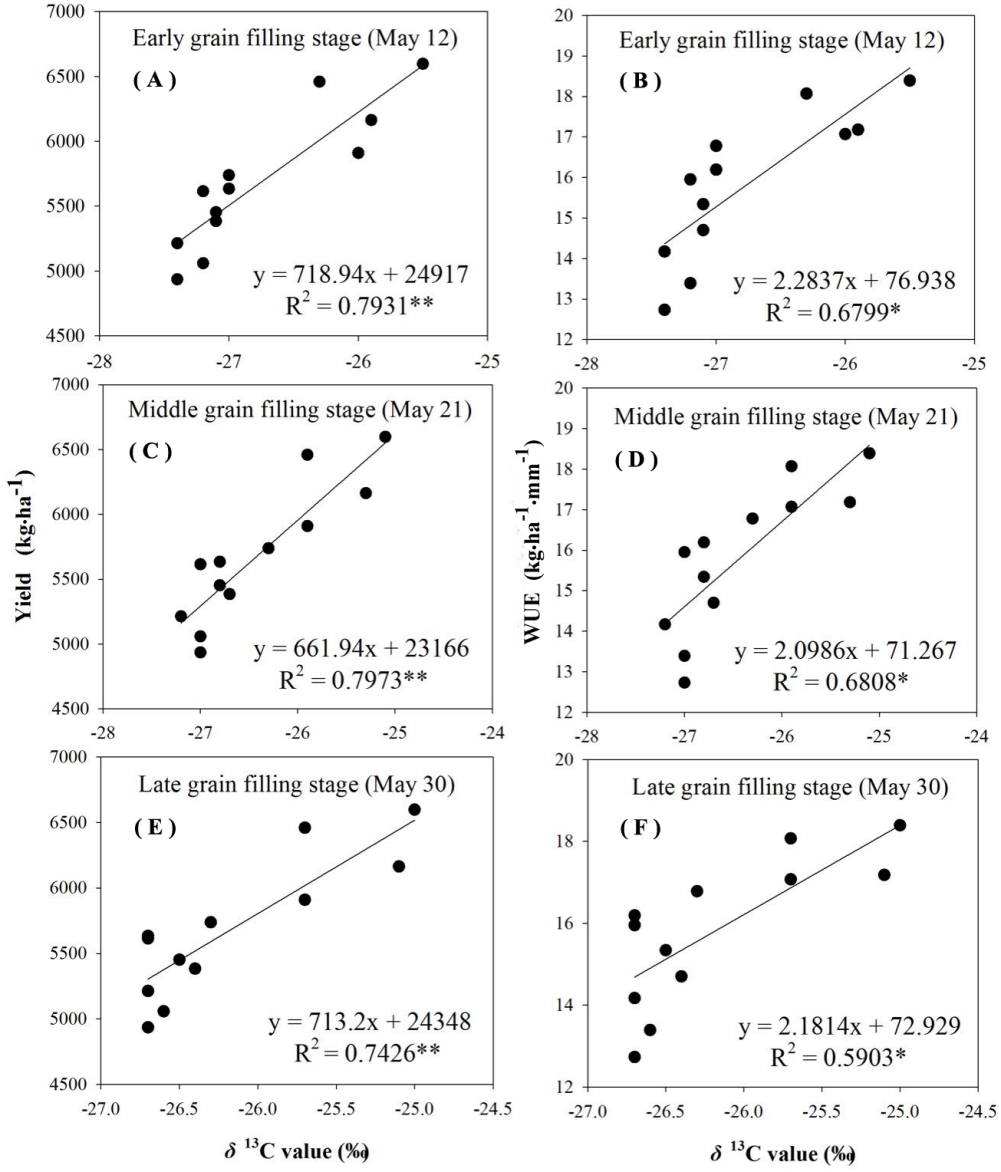
Regressions of stable carbon isotope ratio (δ13C) value in wheat flag leaves with grain yield and water use efficiency (WUE) during different grain filling stages under rain-fed condition in 2015–2016. (A) Regression of stable carbon isotope ratio (*δ*^13^C) value in flag leaves of wheat and grain yield at early grain filling stage under rain-fed condition in 2015–2016. (B) Regression of stable carbon isotope ratio (δ13C) value in flag leaves of wheat and WUE at early grain filling stage under rain-fed condition in 2015–2016. (C) Regression of stable carbon isotope ratio (δ13C) value in flag leaves of wheat and grain yield at middle grain filling stage under rain-fed condition in 2015–2016. (D) Regression of stable carbon isotope ratio (δ13C) value in flag leaves of wheat and WUE at middle grain filling stage under rain-fed condition in 2015–2016. (E) Regression of stable carbon isotope ratio (δ13C) value in flag leaves of wheat and grain yield at late grain filling stage under rain-fed condition in 2015–2016. (F) Regression of stable carbon isotope ratio (δ13C) value in flag leaves of wheat and WUE at late grain filling stage under rain-fed condition in 2015–2016.

**Figure 5:**
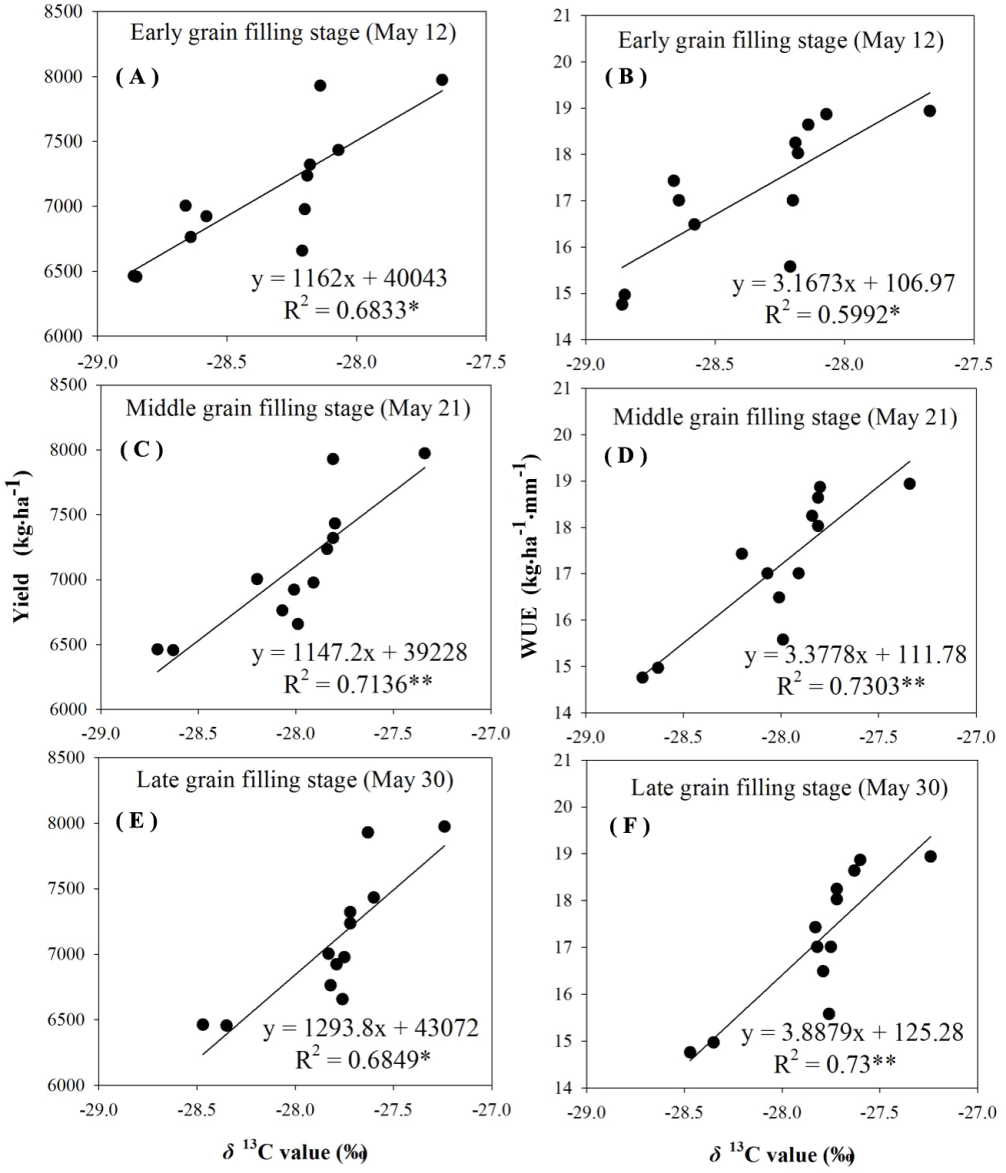
Regressions of stable carbon isotope ratio (δ13C) value in wheat flag leaves with grain yield and water use efficiency (WUE) during different grain filling stages under supplement irrigation condition in 2015–2016. (A) Regression of stable carbon isotope ratio (δ13C) value in flag leaves of wheat and grain yield at early grain filling stage under supplemental irrigation condition in 2015–2016. (B) Regression of stable carbon isotope ratio (δ13C) value in flag leaves of wheat and WUE at early grain filling stage under supplemental irrigation condition in 2015–2016. (C) Regression of stable carbon isotope ratio (δ13C) value in flag leaves of wheat and grain yield at middle grain filling stage under supplemental irrigation condition in 2015–2016. (D) Regression of stable carbon isotope ratio (δ13C) value in flag leaves of wheat and WUE at middle grain filling stage under supplemental irrigation condition in 2015–2016. (E) Regression of stable carbon isotope ratio (δ13C) value in flag leaves of wheat and grain yield at late grain filling stage under supplemental irrigation condition in 2015–2016. (F) Regression of stable carbon isotope ratio (δ13C) value in flag leaves of wheat and WUE at late grain filling stage undersupplemental irrigation condition in 2015–2016.

**Figure 6:**
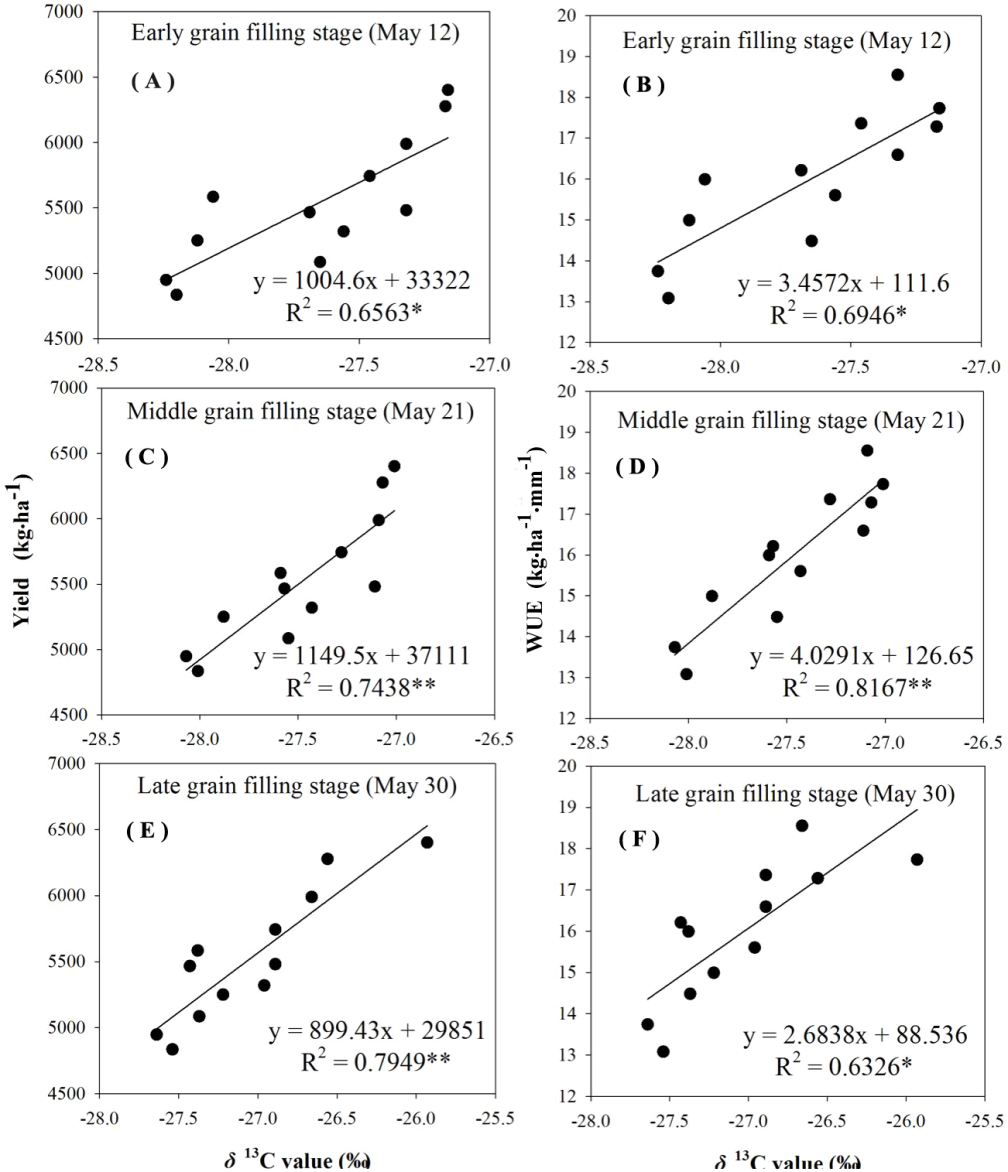
Regressions of stable carbon isotope ratio (δ13C) value in wheat flag leaves with grain yield and water use efficiency (WUE) during different grain filling stages under rain-fed condition in 2016–2017. (A) Regression of stable carbon isotope ratio (δ13C) value in flag leaves of wheat and grain yield at early grain filling stage under rain-fed condition in 2016–2017. (B) Regression of stable carbon isotope ratio (δ13C) value in flag leaves of wheat and WUE at early grain filling stage under rain-fed condition in 2016–2017. (C) Regression of stable carbon isotope ratio (δ13C) value in flag leaves of wheat and grain yield at middle grain filling stage under rain-fed condition in 2016-2017. (D) Regression of stable carbon isotope ratio (δ13C) value in flag leaves of wheat and WUE at middle grain filling stage under rain-fed condition in 2016–2017. (E) Regression of stable carbon isotope ratio (δ13C) value in flag leaves of wheat and grain yield at late grain filling stage under rain-fed condition in 2016–2017. (F) Regression of stable carbon isotope ratio (δ13C) value in flag leaves of wheat and WUE at late grain filling stage under rain-fed condition in 2016–2017.

**Figure 7:**
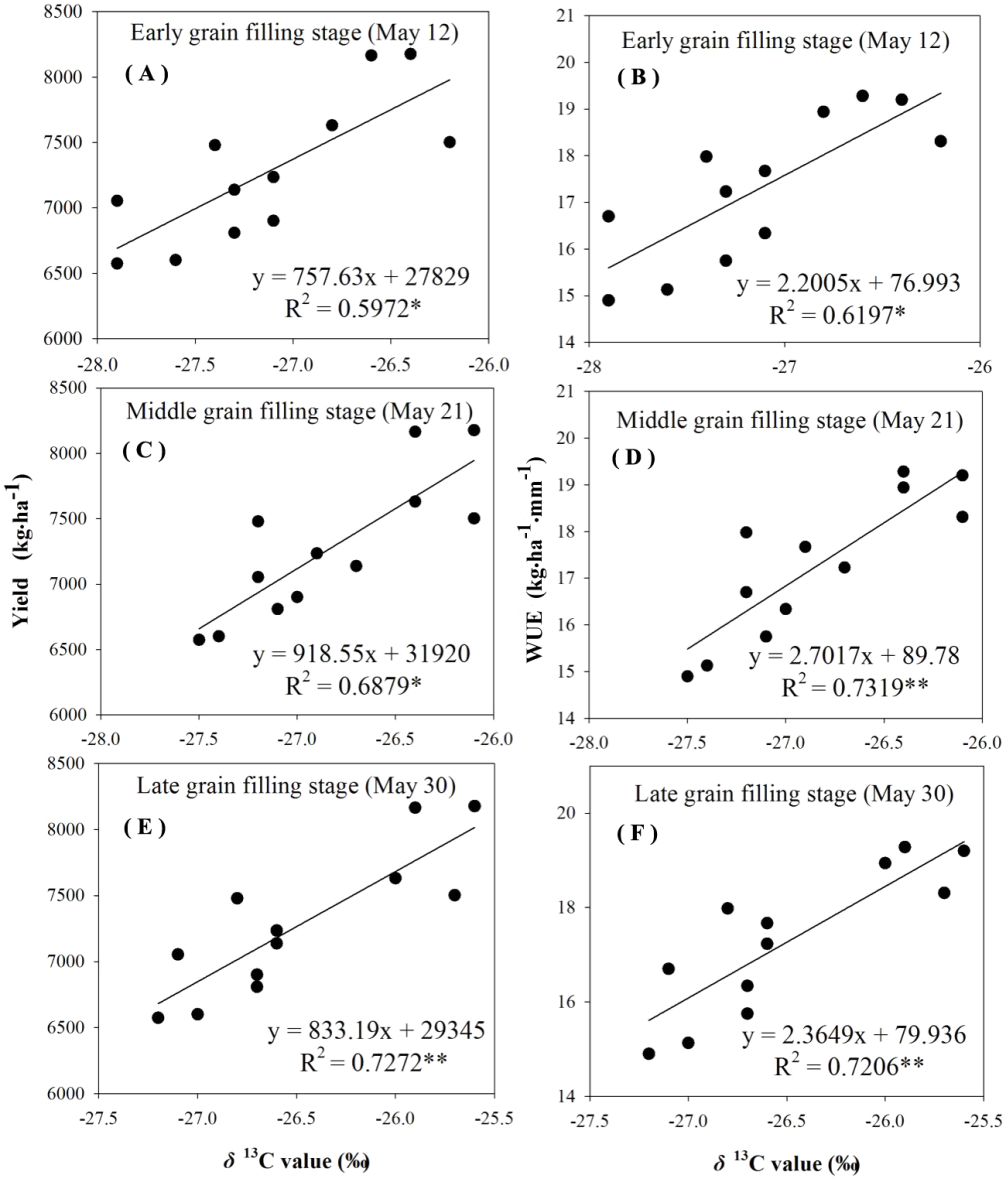
Regressions of stable carbon isotope ratio (δ13C) value in wheat flag leaves with grain yield and water use efficiency (WUE) during different grain filling stages under supplement irrigation condition in 2016–2017. (A) Regression of stable carbon isotope ratio (δ13C) value in flag leaves of wheat and grain yield at early grain filling stage under supplemental irrigation condition in 2016–2017. (B) Regression of stable carbon isotope ratio (δ13C) value in flag leaves of wheat and WUE at early grain filling stage under supplemental irrigation condition in 2016–2017. (C) Regression of stable carbon isotope ratio (δ13C) value in flag leaves of wheat and grain yield at middle grain filling stage under supplemental irrigation condition in 2016–2017. (D) Regression of stable carbon isotope ratio (δ13C) value in flag leaves of wheat and WUE at middle grain filling stage under supplemental irrigation condition in 2016–2017. (E) Regression of stable carbon isotope ratio (δ13C) value in flag leaves of wheat and grain yield at late grain filling stage under supplemental irrigation condition in 2016–2017. (F) Regression of stable carbon isotope ratio (δ13C) value in flag leaves of wheat and WUE at late grain filling stage undersupplemental irrigation condition in 2016–2017.

The reliability of using δ^13^C to characterize *WUE* and grain yield was examined by analyzing the correlation between δ^13^C values, grain yield, and *WUE*. There was a linear positive significant correlation between the *δ*^13^C values, the grain yield, and *WUE* during different grain-filling periods under rain-fed and supplemental irrigation conditions (Figs. 4–7). The *δ*^13^C values increased during early grain-filling stage to the late grain-filling stage (Tables 6–7). However, the changes in the δ^13^C values did not cause changes in the linear relationships between δ^13^C values, the grain yield, and *WUE* (Figs. 4–7). Whether under rain-fed conditions or supplemental irrigation conditions, the genotypes with high *δ*^13^C values exhibited higher grain yield and *WUE*. On the contrary, the grain yield and *WUE* in genotypes with lower δ^13^C values were relatively lower. This indicated that the *δ*^13^C value measurement in winter wheat can be performed at any time during the grain-filling period without affecting its correlation with *WUE* and grain yield.

## Discussion

In practical terms, *WUE* at the field population scale deserves the most attention rather than the *WUE* at leaf level and the individual or intrinsic *WUE* (Mei et al., 2013). As well known, however, evaluation of *WUE* at the field population scale is laborious, time consuming, thus impossible to evaluate a large number of materials. The present study explored how the easily measured trait δ^13^C correlated with field population *WUE* in 12 selected winter wheat genotypes under rain-fed and supplemental irrigation treatments. This might provide helpful information for finding reliable surrogate traits for *WUE* evaluation.

### Effects of different factors on *WUE* and *δ*^13^C

The results indicated that there were significant differences in grain yield and *WUE* among winter wheat genotypes. Grain yield and *WUE* were higher under supplemental irrigation conditions in comparison with those under rain-fed conditions. These were consistent with previous researches (Li et al., 2014). The lower *WUE* of different genotypes under rain-fed conditions was mainly due to the fact that less water supplied during the experimental years and a severe degree of drought stress subsequently affected plant growth. Midgley & Moll (1993) conducted research on the cyclic drought stress in perennial shrubs in South Africa and pointed out that *WUE* increased with moderate water content of the soil, and when the soil water content was too low, stomatal conductance became conservative and eventually stabilized, and *WUE* began to decline again.

Previous studies have also shown that there are significant differences in δ^13^C values in C_3_, C_4_, and CAM plants (Mook, Bommerson & Staverma, 1974; O’Leary, 1984; Farquhar, Ehleringer & Hubick, 1989). Due to the synergistic effect of the absorption and diffusion phases in the enzymatic reaction during CO_2_ formation from carboxyl groups after isotope fractionation, the same photosynthetic pathway plants also exhibit the phenomenon of high degree of differentiation. The 12 winter wheat genotypes belonged to C_3_ plants, and the δ^13^C values in flag leaves in winter wheat genotypes exhibited significant differences for 3 measurements. The above theories sufficiently explain the results of the present study. Our study also showed that δ^13^C values slowly increased during the three measurements, whether under rain-fed conditions or supplemental irrigation at the jointing and anthesis stages. After plants develop into grain filling stage, strong photosynthesis for rapid accumulation of assimilates began to recede gradually. Instead, distribution and conversion of assimilates to grain turn to predominate this stage. The weakened photosynthesis leads to increased Ci/Ca, which in turn result in reduction of CO_2_ entering into mesophyll cells. At this time, the ^13^C could not be fractionated in a timely fashion by the carboxylase of the plants, and then organic matter was directly synthesized with high δ^13^C values (Van de Water, Leavitt & Betancourt, 1994; Feng & Epstein, 1995).

As shown by previous studies, water stress induce stomatal closure, then cause decrease in stomatal conductance and Ci, which in turn leads to an increase in the δ^13^C values of fixed carbon in plant photosynthesis. Conversely, a decrease in δ^13^C values comes out under more water and high nutrient conditions. The δ^13^C values for the different genotypes of wheat were higher under rain-fed conditions than that under corresponding irrigation conditions. The differences in the δ^13^C values among the genotypes in this experiment were greater than that between different measurement periods and different water treatments (Tables 6–8), which indirectly indicates that the δ^13^C values in flag leaves of winter wheat have relative stability. Dramatically, the factor investigation year had the largest effect on δ^13^C, differing from its influence on yield, water consumption, and WUE. δ^13^C was affected by intrinsic photosynthetic capacity, driven by the amount of carboxylating enzyme (Bort et al., 2014; Condon et al., 2002). Precipitation in the wheat grain filling stage of 2016–2017 growing season was larger than that in 2015–2016 growing season (Fig. 1). Plant photosynthesis sensitively responds to water status, as well known. Thus we would rather ascribe the substantial influence of year on δ^13^C to the precipitation difference in grain filling stage between the two investigation years.

### Correlations between *δ*^13^C and *WUE*

The negative relationship between carbon isotope discrimination and *WUE* at leaf level for wheat genotypes was first proposed in 1984 (Farquhar & Richards, 1984). In the current study, the δ^13^C values in leaf material showed a significant linear positive correlation with *WUE* at field population level under the experimental conditions (Figs. 4–7). The correlation between δ^13^C values during the three determination periods at the grain-filling stages with grain yield and *WUE* reached an extremely significant level, and different water conditions had no effect on the correlation between the δ^13^C values with grain yield and *WUE*, which is consistent with a large number of previous studies (Wright, Rao & Farquhar, 1994; Condon, Richards & Rebetzke, 2004). The data implied that comparisons might be carried out at any time during the grain-filling stage under various moisture conditions to indicate *WUE* in winter wheat with δ^13^C values as an indicator.

The theoretical basis of the linear positive correlation between the δ^13^C values and leaf *WUE* can be obtained from the calculation formula of δ^13^C values and *WUE* derived from Farquhar, O’Leary & Berry (1982) and Condon, Richards & Rebetzke (2004). Their researches indicate that plant δ^13^C values and *WUE* have the same influencing factor of Ci/Ca. Therefore, *WUE* can be deduced by measuring the plant *δ*^13^C values. However, there are also reports that the δ^13^C values are negatively correlated or not correlated with yield and *WUE* (Delucia & Schlesinger, 1991; Fengjun et al., 2006; Chamekh et al., 2016). This might be ascribed to the differences in water conditions and growth environments. It can be seen that the degree of water stress may have an effect on the correlation between δ^13^C values with grain yield and *WUE*, although the specific reasons require further study. Therefore, it is necessary to define water conditions clearly for using δ^13^C value as surrogate trait in screening water-saving crop varieties.

In addition to the effect of water conditions discussed above, the correlation between δ^13^C and *WUE* seems to be related with crop species and temporal-spatial scale of *WUE.* Transpiration efficiency (TE), different from field *WUE* in scale, is biomass accumulation divided by water consumed through transpiration of individual plant in a period of time. In legume plants, TE was reported to negatively correlate or have no correlation with δ^13^C. A research conducted in the International Crops Research Institute for the Semi-Arid Tropics (ICRISAT) found TE was negatively associated with △^13^C in groundnut leaves at the final harvest after drought stress imposition, but fairly weak (*R*^2^ = 0.13) (Krishnamurthy et al., 2007). In lentil (*Lens culinaris* Medikus), chickpea (*Cicer arietinum* L.), and lupin (*Lupinus angustifolius* L.) 3 grain legumes, leaf δ^13^C and TE in the vegetative phase were not significantly correlated under well-watered conditions *(Turner et al., 2007*). Also, no correlation between TE and leaf △^13^C in groundnut isogenic materials with large variation in TE was found regardless of the water regime (Devi et al., 2011). In wheat, however, positive correlations between △^13^C and field *WUE* or yield are apt to be reported more by the researches. Leaf △^13^C at anthesis positively correlated with grain yield for spring wheat in the post-anthesis water stress conditions of northwest Mexico. Both leaf △^13^C and grain △^13^C were strongly and positively correlated to grain yield under rain-fed conditions of northern China (Xu et al., 2007). These are in agreement with our results in winter wheat. Therefore, to elucidate and then efficiently use the relations between δ^13^C and *WUE* in screening water saving varieties, crop species and *WUE* scale need to be fully considered.

In general, though the accumulating studies proved high stability of δ^13^C characterizing plants, the relationship between δ^13^C value and *WUE* were determined by diverse environmental factors, crops species, along with *WUE* scale. Therefore, in order to improve the reliability and scientific accuracy of the δ^13^C value as an indicator to assist with breeding water-saving genotypes, further studies need to be performed to elucidate some definite relations under specific environmental conditions.

## Conclusions

Leaf δ^13^C values at grain-filing stage significantly correlated with *WUE* of field population level in both rain-fed and irrigation status under the climate conditions of the northwest Huang-Huai-Hai Plain, as proved by our experiments conducted in two consecutive growing seasons. This may imply that leaf δ^13^C of grain filling stage might be a potential useful surrogate trait for field population *WUE*, which is of practical guidance but of difficulty in evaluation. Referring to the previous relative studies, the relations between leaf δ^13^C and *WUE* are considered to depend on multiple factors, such as soil water status, climate conditions, crop species, etc. Therefore, to improve the reliability of δ^13^C value as an indicator to assist with breeding and selecting water-saving cultivars, further studies need to be performed. Enlarged number of wheat materials, experimental sites under different climate, and different soil water regimes all remain to be taken account. To conclude, for obtaining definite correlations between *δ*^13^C and population *WUE*, it is absolutely essential to specify environmental conditions by conducting further systematic researches.

##  Supplemental Information

10.7717/peerj.6767/supp-1 Dataset S1 Data for tables and figures Date for tables and figures including grain yield, water consumption, WUE and *δ*^13^C.

## Additional Information and Declarations

### Data Availability

The following information was supplied regarding data availability: The raw data are available in a Supplemental File.
